# Use of portable air purifiers to reduce aerosols in hospital settings and cut down the clinical backlog

**DOI:** 10.1017/S0950268823000092

**Published:** 2023-01-18

**Authors:** Jacob Salmonsmith, Andrea Ducci, Ramanarayanan Balachandran, Liwei Guo, Ryo Torii, Catherine Houlihan, Ruth Epstein, John Rubin, Manish K. Tiwari, Laurence B. Lovat

**Affiliations:** 1 Department of Mechanical Engineering, University College London, London, UK; 2 Rare and Imported Pathogens Laboratory, UK Health Security Agency, London, UK; 3 Department of Infection and Immunity, University College London, London, UK; 4 Department of Otolaryngology, Royal National Ear Nose and Throat and Eastman Dental Hospital, University College London Hospitals NHS Foundation Trust, London, UK; 5 Division of Surgery & Interventional Science, University College London, London, UK; 6 Wellcome/EPSRC Centre for Interventional & Surgical Sciences (WEISS), University College London, London, UK; 7 Gastrointestinal Services, University College London Hospital, London, UK

**Keywords:** COVID-19, hygiene and hospital infections, infectious disease control, prevention, public health

## Abstract

SARS-CoV-2 has severely affected capacity in the National Health Service (NHS), and waiting lists are markedly increasing due to downtime of up to 50 min between patient consultations/procedures, to reduce the risk of infection. Ventilation accelerates this air cleaning, but retroactively installing built-in mechanical ventilation is often cost-prohibitive. We investigated the effect of using portable air cleaners (PAC), a low-energy and low-cost alternative, to reduce the concentration of aerosols in typical patient consultation/procedure environments. The experimental setup consisted of an aerosol generator, which mimicked the subject affected by SARS-CoV-19, and an aerosol detector, representing a subject who could potentially contract SARS-CoV-19. Experiments of aerosol dispersion and clearing were undertaken *in situ* in a variety of rooms with two different types of PAC in various combinations and positions. Correct use of PAC can reduce the clearance half-life of aerosols by 82% compared to the same indoor-environment without any ventilation, and at a broadly equivalent rate to built-in mechanical ventilation. In addition, the highest level of aerosol concentration measured when using PAC remains at least 46% lower than that when no mitigation is used, even if the PAC's operation is impeded due to placement under a table. The use of PAC leads to significant reductions in the level of aerosol concentration, associated with transmission of droplet-based airborne diseases. This could enable NHS departments to reduce the downtime between consultations/procedures

## Introduction

**Fig. 1.**
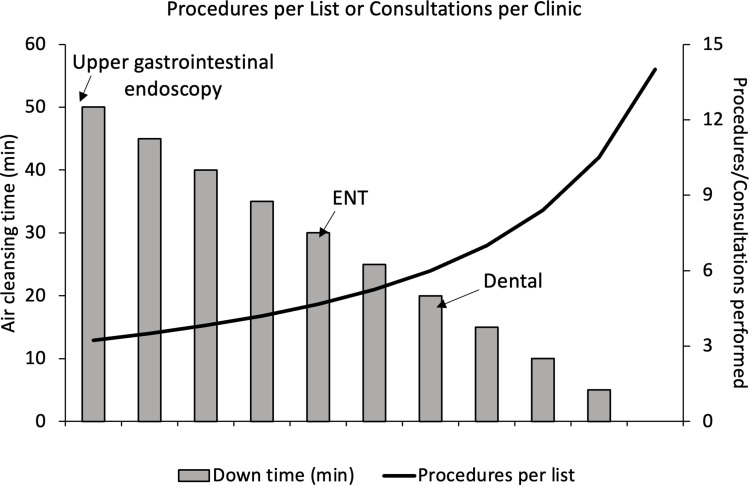
Rising healthcare backlogs. Effect of air cleansing downtime between procedures/consultations upon the number of 15 min procedures/consultations possible in a 3.5 h session at University College Hospital (UCLH) in London, November 2021 (these vary by hospital). Specific air cleansing recommendations for upper gastrointestinal endoscopy, ear nose and throat (ENT), and dental procedures/consultations are indicated.

The highly infectious nature of SARS-CoV-2 has severely impacted the day-to-day operation of hospitals. Early experiences with patient-to-patient and patient-to-staff infections combined with a lack of robust data in how to reduce the risk of infectious air have led to a conservative approach with large fallow periods in usually busy procedure/consultation clinics (Fig. 1). Some departments have been more heavily affected than others, such as ear nose throat (ENT), gastroenterology and dental [1], one of the reasons being the involvement of aerosol generating procedures (AGPs). Typical AGPs in those departments include sinus surgery, routine suctioning or dental drilling, which have not been well characterised and are highly variable in terms of their aerosolising potential; as are coughing or sneezing induced by flexible nasendoscopy, a common and routine ENT outpatient procedure [2–7]. Additionally, the aerosol and droplet characteristics resulting from common human breathing, speaking, shouting or coughing vary substantially from person to person [8]. Droplets < 100 μm tend to evaporate after leaving the respiratory air chambers and remain suspended in the air, behaving similarly to aerosols [9]. No unified measurement approach yet exists for aerosols and droplets < 100 μm [3, 10], but recent evidence suggests that aerosols and small droplets produced by patients in general, not just AGPs, may be the most important mode of transmission [7, 11–15]. Regardless, the need for inexpensive and bespoke mitigations against virus spreading via aerosols and small droplets is clear.

Incorporating built-in mechanical ventilation systems into existing hospitals and other healthcare facilities is often prohibitively expensive and requires significant downtime, further increasing already swollen patient waiting lists – in July 2022 over 6.6 million patients are waiting for National Health Service (NHS) treatment in the UK [16]. Over 330 000 patients have been waiting for more than 1 year, 13 times the number from May 2020. Even for buildings that already have built-in filtered mechanical ventilation, this may not be sufficient to filter out virus particles, even when using filter grades as high as MERV 13 or 14 [17]. Furthermore, built-in mechanical ventilation has been shown to potentially spread, rather than inhibit, pathogenic particles as soon as 20 days after being cleaned [18]. High-efficiency particulate air (HEPA) filters have been shown to be effective in removing infectious SARS-CoV-2 particles of all sizes from the air [19–21]. Use of portable air cleaners (PAC) with HEPA filters also contribute to the air changes in a room, improving ventilation and reducing build-up of infectious particles [17, 22, 23]. They also impose lower energy footprint for the building compared to built-in mechanical ventilations. Thus, the use of easily and affordably managed PACs may be an effective strategy to reduce risk of aerosol transmission when built-in mechanical ventilation is not available or is partially effective [6]. Similarly, a PAC placed between patient and doctor may further reduce aerosol transmission of airborne viruses than use of built-in mechanical ventilation alone. A deeper investigation into the use of PAC to mitigate the risk of airborne diseases is clearly an important step in reducing the transmission of SARS-CoV-19 and other airborne diseases.

In this paper, we report our investigation on the effect of using PACs to reduce the levels of small aerosols in a room after the aerosol source has been removed. Faster removal of suspended aerosols from the air could reduce downtime between hospital consultations and procedures, increasing the number of patients seen, and helping to tackle hospital waiting lists. Figure 1 shows actual down times that were implemented at UCLH for different AGP types during the pandemic for patients who were not proven to be negative for SARS-CoV2 infection. These times were based on a combination of the generalised rates of air change of the existing built-in mechanical ventilation and the department type (e.g. gastroenterology, ENT and dental) where the room was located.

## Methods

We developed an experimental setup consisting of an aerosol generator, which mimicked the subject affected by SARS-CoV-19, and an aerosol detector, reflective of recipient exposure to SARS-CoV-19. Using this system, experiments of aerosol dispersion and clearing were undertaken *in situ* in a variety of rooms (i.e. laboratory, procedure and consultation rooms), with a layout consistent with reported practices of procedure/consultation rooms.

### Aerosol generation

Aerosols were introduced into the environment by attaching a 1 bar air pressure supply to a nebuliser (CompAIR C28P, Omron, Japan) filled with saline (9 g NaCl/1 litre deionised water). Saline was used to more closely match the evaporation characteristics of saliva than would result from using pure water [7]. The outflow from the nebuliser was then guided through an android-like construction (referred to as ‘VALUATOR’), and ejected from a rectangular opening at a height of 1.2 m, equivalent to the mouth of a seated person, seeding the surrounding air with saline aerosols with a mass median aerodynamic diameter of 3 μm, consistent with our previous studies on human aerosol production [8]. The outlet was positioned at a height consistent with a sitting person's (e.g. a patient) mouth while having a conversation. The volume flow rate was measured with a flowmeter (MASS-VIEW MV-308, Bronkhorst, the Netherlands), positioned in between the nebuliser and outlet from VALUATOR. A typical layout of the aerosol generator and detector is provided in the Supplementary information (Fig. S1).

The operating conditions of VALUATOR were established by combining preliminary laser analysis experiments (see Supplementary data) to ensure the velocity range of exiting aerosols matched the velocity of particles breathed out by a range of human volunteers in a previous study [8], where median aerosol velocity ranged from 0.47 to 0.78 m/s across a range of vocal tasks. In the current experiments, a flow rate of 2.5–2.7 litres/m was selected, which corresponded to a mean aerosol velocity of 0.45 m/s, with a peak velocity of 1.19 m/s. This aerosol exit velocity was periodically monitored during each experiment with a hot wire anemometer (RS-8880, RS Group, UK) taken from the mouth of VALUATOR. A baseline of aerosol concentration was measured using the aerosol detection system described below before any seeding, with the baseline defined as the mean of five consecutive pre-seeding measurements. Aerosolised saline aerosols were then generated at a constant rate into the room (unless specifically noted) until a filling steady-state aerosol concentration (FSSAC) was achieved. This was defined as when the aerosol concentration readings remained constant ±10% for five consecutive readings (i.e. over a 10 min period). Once FSSAC had been achieved, the air/aerosol supply was switched off, and aerosol concentration was regularly measured with 2 min interval during the emptying phase until the concentration level returned to the baseline measured before aerosol generation.

### Aerosol detection

Aerosols were detected and quantified using a laser spectrometer (Aerodynamic Particle Sizer (APS) 3321, TSI Incorporated, MN, USA). The APS operated under standard manufacturer's settings, at a flow rate of 5 litres/m ± 0.2 but only 1 litres/m of this flow partitioned off, sized and counted. The APS measures the volumetric concentration (cm^−3^) and size distribution of aerosols with diameter < 19 μm. Air samples were taken for 60 s every 120 s. A 1 m length of 25 mm section PVC hosing was attached to the inlet nozzle of the APS, with a plastic funnel fixed to the other end of the hose. The inlet to this funnel was placed at 1.2 m, where a second seated person's (e.g. the consultant/medical practitioner's) mouth would be expected to be during a conversation.

### Rooms

**Fig. 2.**
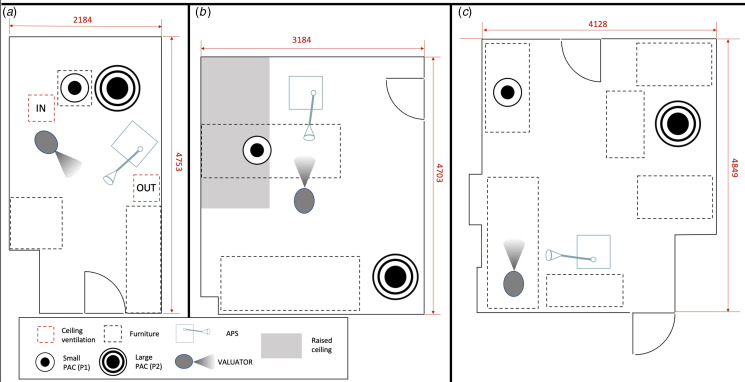
Room schematics. (a) UCL laboratory room (R1) – IN and OUT indicate position of built-in mechanical ventilation in- and out-flow. (b) Consultation room, Chandler Wing, UCLH (R2). (c) Consultation/procedure room, Albany Wing, UCLH (R3).

The effect of aerosol mitigation strategies was analysed in three different rooms in this study: a laboratory room at UCL (R1), measuring approximately 2.1 m × 4.5 m × 4.0 m, with inlet and outlet air ventilation panels (Fig. 2a); and two rooms in the National Hospital for Neurology and Neurosurgery, part of University College London Hospitals NHS Foundation Trust (UCLH). The two hospital rooms were chosen from this Victorian building as they have no ventilation and represent many similar rooms throughout old hospitals which are still widespread in the NHS [24]. The first room was a consulting room (R2), measuring approximately 3.2 m × 4.7 m × 2.6 m, with no ventilation panels (Fig. 2b); and the second was a procedure room (R3), measuring approximately 4.1 m × 4.8 m × 2.5 m, with no ventilation panels (Fig. 2c).

Experiments were run in room R1 with the built-in mechanical ventilation either switched on or off. A coarse estimation of the inflow and outflow to the room was obtained by assuming a constant velocity measured with the hot-wire anemometer across the ventilation system inlet/outflow surface areas. This led to an estimate of an inflow of 3–4 m^3^/min, an outflow through the vent of 1–2 m^3^/min, with remaining outflow occurring around the door and various pipe joints at the top of the room, resulting in approximately 5.4 air changes/h. Neither R2 nor R3 had any built-in mechanical ventilation.

### Mitigation type and placement

Two different PACs were used in this study, representative of two typical and cheap PACs: a smaller unit, P1 (Core 200S Smart True HEPA Air Purifier, Arovast Corporation, CA, USA), with a maximum clean air delivery rate (CADR) of 200.6 m^3^/h; and a larger unit, P2 (LV-H133 Tower True HEPA Air Purifier, Arovast Corporation) with CADR of 466 m^3^/h when used on the maximum setting. When switched on, devices P1 and P2 were set to medium (setting 2 of 3), resulting in a flow rate of 1.1 m^3^/min (equivalent to a CADR of 68.9 m^3^/h) and 2.2 m^3^/min (equivalent to a CADR of 155.3 m^3^/h), respectively. The medium flow setting was chosen after consultation with medical professionals, who indicated that while the high flow setting was too noisy to be used during all conversations with patients, the medium flow setting was acceptable. Further details for P1 and P2 can be found in the supplemental data for this paper. Experiments with no PACs switched on are indicated by ‘NP’.

P1_i_ and P2_i_ were ‘ideal’ placements of the PAC, defined as having a relatively unobstructed flow of air to and from the PAC, as well as being at a raised height of ~1 m in the case of P1. ‘Non-ideal’ placements of air purifiers reported to us by hospital staff during preliminary consultations are denoted as follows: under a table (P1_t_), pushed into the corner of the room (P1_c_ and P2_c_) or placed by the door (P1_d_). Further PAC placement details are given in Table 1. Due to time constraints in the hospital setting, a thorough investigation of PAC positions was carried out in the laboratory setting (R1), while only the NP, ideal and worst case (PAC under a table) PAC position scenarios were investigated in the hospital rooms (R2 and R3).

**Table 1.** Mitigation position and orientation

Mitigation setup	Height of mitigation base from ground (m)	Mitigation distance from VALUATOR (m)	Notes
P1_i_	0.8	0.5	
P1_t_	0.0	>2.0	Tabletop height of 0.8 m
P1_c_	0.0	>2.0	PAC rotated so inlet area obstructed
P1_d_	0.0	>2.0	
P2_i_	0.0	>1.0	
P2_c_	0.0	>2.0	PAC rotated so inlet area obstructed

Mitigation strategies analysed for each room were (NB – vents were sealed off unless noted):

- R1 – NP with open vents; no mitigation; P1_i_ with open vents; P1_i_; P1_t_; P1_d_; P1_c_; P2_i_; P2_c_; P1_i_ and P2_i_
- R2 – NP; P1_i_; P1_t_; P2_i_; P1_i_ and P2_i_
- R3 – NP; P1_i_; P1_t_; P2_i_; P1_i_ and P2_i_

### Aerosol concentration analysis

To analyse the temporal variation of aerosol concentration in each room, the time history of aerosol concentration was fit to a basic mathematical model assuming a well-mixed concentration throughout the entire room at any given time, in line with previous studies on indoor airborne virus transmission [23]. The model assumed FSSAC at the end/beginning of the filling/decay stage, with the mitigation acting as a sink. The aerosol removal is referred as decay (reflecting concentration decay). This results in a first-order differential equation, which is solved by an exponential decay function for the aerosol removal stage as follows:1
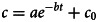
where *c* = aerosol concentration (#/cm^3^), *t* = time (minutes), and *a*, *b* and *c*_0_ are constants specific to each decay curve (in the units #/cm^3^, min^−1^ and #/cm^3^ respectively). When finding the coefficients of Equation 1 through a non-linear regression fit, the emptying steady-state aerosol concentration constant, ‘*c*_0_’, was set to assume only positive values. The FSSAC is equal to *a* + *c*_0_ and constant *b* is referred to in this paper as ‘the decay rate’. The half-life of the aerosol cleaning was calculated using Equation 2:2
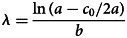
where λ is in minutes. λ is the time required for a specified property, in this case the detected aerosol concentration, to decrease by half.

### Validation of experimental protocol

The nebuliser setup produced aerosols at much higher rate (>2200/cm^3^/s) than two people sitting down and talking to each other (<4/cm^3^/s [7]). This had benefits, such as increasing the signal: noise due to ambient airborne-particles, measured as having an initial baseline aerosol concentration of 6.34 ± 1.05/cm^3^. However, a further validation experiment was necessary, to assess the impact of using different levels of initial aerosol volumetric concentrations on the decay rate and λ. The details of this validation are presented in the Supplementary information and concluded that the proposed experimental protocol increases the signal:noise error and provides a conservative estimate of air cleaning time.

As time in the hospital was limited, only one run of each setup would be undertaken, in order to test for a wide variety of mitigation types and configurations. To increase confidence that a single run would be representative of a specific mitigation type/configuration and would contribute towards valid comparisons between the various setups, multiple runs of the same mitigation type/configuration were run in the UCL lab, to see the variability of the decay constant and half-life for each setup.

Each of the chosen two setups – no PAC with closed mechanical ventilation and PAC P1 with closed mechanical ventilation – was run three times, with the results shown in Supplementary Table S2. For the no PAC-closed vents setup, this gave a decay constant of 0.0316–0.0330 and a half-life of 21.2–22.1 min, i.e. a range of 4.4% and 4.2% respectively. For the P1 PAC-closed vents setup, this resulted in a decay constant of 0.0865–0.0911 and a half-life of 7.8–8.1 min, i.e. a range of 5.3% and 3.8% respectively.

## Results

### Laboratory setting

**Fig. 3.**
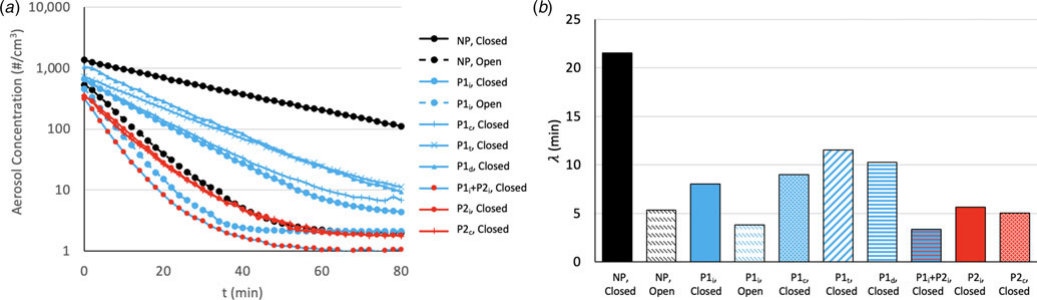
Variation of decay with mitigation setups. (a) Reduction of aerosol concentration with time for various mitigations strategies starting from ‘full’ steady state. (b) Half-life (λ) for each mitigation type. All PACs were set to medium when used. ‘Open’ or ‘Closed’ refers to the inlet/outlet of the mechanical ventilation in the ceiling. ‘_i_’ (ideal), ‘_c_’ (corner), ‘_t_’ (table) and ‘_d_’ (door) refer to the position of the active portable air cleaner (PAC). NP, no PAC; P1, small PAC on medium setting; P2, large PAC on medium setting.

Decay curves and λ for R1 are displayed in Figure 3 for all mitigation setups.

FSSAC was achieved for each mitigation setup, with FSSAC values equal to the aerosol concentration at time 0 in Figure 3a. Baseline aerosol concentration and FSSACs are reported in the Supplementary information for all setups. Decay curve analysis yielded λ ranging from 3.4 min (P1_i_ + P2_i_) to 21.6 min (NP)

(Fig. 3b). Non-ideal placement of a small PAC (e.g. P1_t_) results in a 47% decrease in λ (21.6–11.5 min), whilst ideal placement (P1_i_) results in a 63% decrease (21.6–8.0 min). Use of an ideally placed large PAC in addition to an ideally placed small PAC in R1 resulted in a λ reduction of 82% (21.6–3.8 min). The P1_i_ configuration results in a FSSAC of 660 aerosols/cm^3^ compared to 1356 aerosols/cm^3^ for the NP configuration, a decrease of 51%. When placed under a table, use of the P1 PAC resulted in a 46% reduction in FSSAC compared to the NP setup.

### Hospital setting

**Fig. 4.**
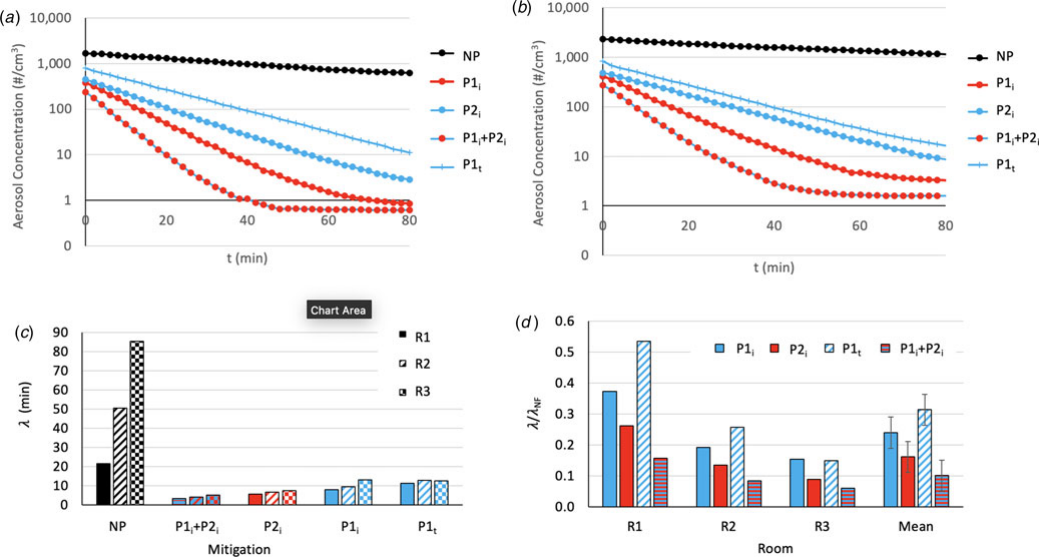
Variation of decay with mitigation setups in hospital rooms. All portable air cleaners (PACs) were set to medium when used, and any mechanical ceiling ventilation was closed. _i_ ‘Ideal’ and _t_ ‘Table’ refer to the position of the active PAC. NP, no PAC; P1, small PAC on medium setting; P2, large PAC on medium setting. Reduction of aerosol concentration with time for various mitigations strategies in (a) UCLH consultation room (R2) and (b) UCLH procedure room (R3). (c) Half-life (λ) for each mitigation type in three different rooms (R1, R2 and R3); (d) Normalised half-life (λ) for each mitigation type grouped by room, λ_NP_ was used to normalise the various λ scores for each room.

Decay curves for aerosol concentration are displayed in Figure 4a for R2 and Figure 4b for R3, for mitigation setups NP, P1_i_, P1_t_, P2_i_ and P1_i_ + P2_i_.

FSSAC was achieved for each mitigation setup, with FSSAC values equal to the aerosol concentration at time 0 in Figures 4a and 4b for R2 and R3, respectively. Baseline aerosol concentration and FSSACs are reported in the Supplementary information for all setups. Decay curve analysis yielded λ ranging from 4.2 min (P1_i_ + P2_i_) to 50.5 min (NP) for R2 and ranging from 5.2 min (P1_i_ + P2_i_) to 85.4 min (NP) for R3 (Fig. 4c). Normalised λ were calculated by dividing the λ for each configuration with the λ for the NP condition (*λ*_NP_), specific to each room (Fig. 4d). The P1_i_ configuration results in a FSSAC decrease of 73% and 79% for rooms R2 and R3 respectively. When placed under a table, use of the P1 PAC resulted in a 53% and 64% reduction in FSSAC compared to the NP setup for rooms R2 and R3 respectively.

### Predicted procedure list aerosol concentrations

**Fig. 5.**
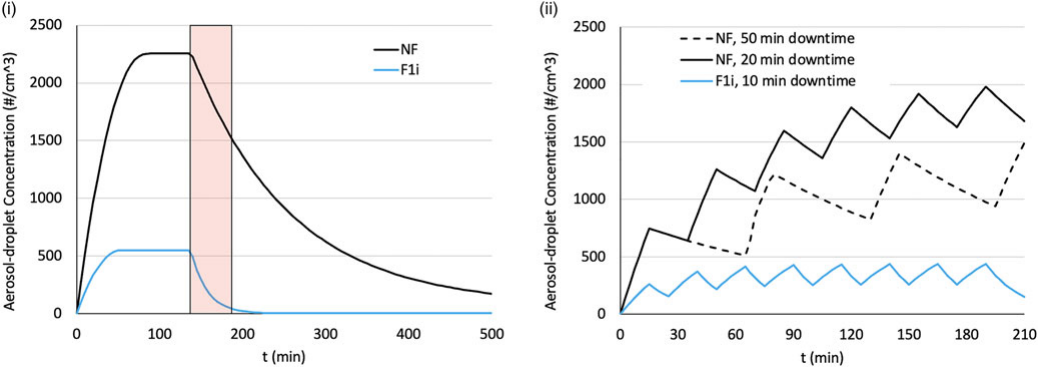
Effect of mitigation upon aerosol concentration. (i) Aerosol concentration curves using either NP or P1i, showing the time to achieve and the magnitude of the filling steady states during filling as well as the time to clean the air once the aerosols are no longer being produced. A 50 min downtime period is indicated by the red rectangle (ii). Aerosol concentration during a typical clinic, with a 15 min procedure time followed by downtime, either using no mitigation (NP) or a small portable air cleaner in a raised location (P1i).

The exponential fit to measured data (Equation 1) can be used for study in other potential scenarios. Using this approach, Figure 5 shows the potential effect of using a small PAC, such as P1, in a typical consultation room, such as R3. Figure 5a shows how the aerosol concentration would typically increase over the course of 2 h, allowing both scenarios to reach FSSAC, with the NP scenario reaching an aerosol concentration over four times that of the P1_i_ scenario. After 50 min of aerosol clearing, the aerosol concentration in the P1_i_ scenario has reduced 92%, whereas the aerosol concentration for the NP scenario has only reduced by 32% and is 280% of the highest concentration predicted for the P1_i_ scenario at any time.

Figure 5b presents characteristic filling and clearing rates predicted for typical consultation/procedure sessions, with 15 min/patient combined with different mitigation strategies in the form of downtime and/or a PAC (P1i).

## Discussion

Figures 3 and 4 clearly indicate that PAC mitigation is very effective in cleaning the air of aerosols in these rooms with minimal other sources of air change, with a significant decrease in aerosol concentration half-life (λ) during decay. Figure 3 shows the effectiveness of built-in mechanical ventilation in comparison to PAC, and the use of PAC P2 (either on its own or in combination with P1) removed aerosols from the air volume at an equivalent or faster rate than mechanical ventilation in R1. This indicates that PAC can be suitable control strategies to further enhance built-in mechanical ventilation and offer a viable alternative when the latter is unavailable due to budgetary, energy or time-sensitive concerns, in agreement with previously stated findings [6]. The implementation of these findings would have a significant impact upon reducing downtime between patients and increasing procedures per list, tackling patient waiting times (Figs 1 and 5a). The viability of the use of a PAC is in agreement with previous research [17, 22, 23]. As HEPA filters have been shown to be effective in removing infectious particles, such as SARS-CoV-2, from the air [19–21], it is reasonable to assume that the reduction in aerosols in the air will result in a reduction in the level of infectiousness in the room [23].

Importantly, the use of mitigations, whether built-in mechanical ventilation or a PAC, during the aerosol generation phase (representing, e.g. when a consultant is talking with a patient) substantially reduces the FSSAC – the highest aerosol concentration that will occur in a room at any given moment. In real-world terms, this means that the level of infectious particles in a room will be kept lower by using even a small PAC, reducing the viral load the occupants in the room will be exposed to, and reducing the chance of infection.

According to Figure 1, UCLH currently requires a downtime of 50 min between upper gastrointestinal endoscopy procedures for patients who have not been proven to be SARS-CoV-2 negative, irrespective of the level of ventilation. Assuming this corresponds to the worst-case scenario of a poorly ventilated room of similar size to R3, 50 min is less than the associated λ (85.4 min). For the same room, the implementation of a small PAC in an ideal location has a λ of 13.2 min, meaning that in the allocated 50 min, the aerosol concentration would be reduced nearly 14-fold. On top of this, the FSSAC utilising P1_i_ is about five times lower than that with no mitigation, further reducing the infectivity risk.

Figure 5b clearly indicates that the use of even one PAC could be more effective in keeping the aerosol concentration in a room below what may result from currently adopted NHS air cleaning strategy – in fact, the aerosol concentration of the P1i scenario throughout the entire session remains below that of either NP scenario after just one procedure. These indications held true irrespective of the room size or layout, although the effect of implanting a PAC was more marked in R3, which had the worst ambient ventilation (Fig. 4d).

### Limitations

We carefully verified potential issues from our experimental procedures. However, we acknowledge there are additional limitations involved in this study. We did not prioritise matching droplet size distribution, humidity, or temperature, due to the focus on dispersion characteristics in these experiments. The use of saline resulted in aerosol size distributions closer to those produced by humans than would have resulted from distilled water, but the use of certain saliva surrogates may have resulted in a closer aerosol size/distribution match. The aerosol generator produced droplets at a constant rate, rather than the more dynamic range that would be produced by a breathing/talking/coughing patient. The impact of saliva surrogates and the installation of programmable low- and high-frequency oscillation (to represent the effect of the diaphragm and the vocal cords respectively) onto the VALUATOR are planned developments to the aerosol generator.

## Conclusion and future work

In this work we show that the use of PACs can markedly reduce the aerosol concentration, accordingly, lowering exposure level of these aerosols to any occupants of the room. This work was carried out in a research laboratory and two hospital consultancy rooms of different sizes. Additionally, the use of PAC may also mitigate the spread of infectious particles from positive pressure rooms, such as operating theatres, to surrounding hospital rooms [25]. Furthermore, as our study is focused on aerosols, the findings from this work can be applied to any pathogens which are spread through aerosols, and is not limited to SARS-CoV-2, suggesting mitigations for future aerosol-transmitted viruses [19]. This work goes some way to addressing the need for robust research on the effectiveness of PAC devices in hospital settings [2, 26, 27], and its implementation would help reduce the crippling patient backlogs without exposing people to increased infection risk and without resulting in significant infrastructure costs. The use of PAC could also be beneficial in other indoor settings besides hospitals where the installation of built-in mechanical ventilation may be cost/time/energy prohibitive, such as offices, schools, universities, etc., as indicated in previous research [28, 29].

Finally, the current study provides a simple and easily deployable method to assess and improve aerosol concentration. This can be used to provide an initial assessment of operating conditions in various hospital rooms. Further, the experimental procedure could be synergistically integrated with detailed computational simulations of the air flow in a room [9, 23, 30], to build a solid platform to tackle this timely and challenging problem.

## Data Availability

Data underlying the figures are available upon request. Requests should be made to the corresponding author.

## Supplementary material

For supplementary material accompanying this paper visit https://doi.org/10.1017/S0950268823000092. click here to view supplementary material
