# Supplementary material for: Use of portable air purifiers to reduce aerosols in hospital settings and cut down the clinical backlog
Source: Epidemiol Infect. 2023 Jan 18;151:e21. doi: 10.1017/S0950268823000092 (PMC9990385; doi:10.1017/S0950268823000092)
Supplement: Supplementary file 1 [file S0950268823000092sup001.docx]

# Use of portable air purifiers to reduce aerosol-droplets in hospital settings and cut down the clinical backlog

## Supplementary Information

Table of Contents

[Supplementary Information 1](#_Toc121824833)

[Experimental Setup 2](#_Toc121824834)

[Portable Air Cleaners 3](#_Toc121824835)

[Laser Measurements 3](#_Toc121824836)

[Validation of experimental protocol 5](#_Toc121824837)

[Experimental Baseline and Filling Steady State Aerosol Concentrations 7](#_Toc121824838)

[Experimental Constants 8](#_Toc121824839)

[Aerosol Size Distributions 10](#_Toc121824840)

[Filling rate 11](#_Toc121824841)

[References 11](#_Toc121824842)

### Experimental Setup

The general experimental setup is shown in SI Figure S1. The nebuliser (f in SI Figure S1) was connected to an inflow of 1 bar pressurised air, and the outflow was routed first through the flowmeter (d) and then through the exit orifice on Sonny (e). This aerosol droplet generator assembly was then placed so that the outlet (e) was located where a patient’s mouth would be expected to be located during a consultation or procedure.

The aerodynamic particle sizer (APS) had a PVC hose connected to the black inlet nozzle on the top of the APS, which in turn had a plastic funnel attached to the inlet end of the PVC hose, forming the aerosol droplet detector setup. This funnel was then placed where the medical professional’s mouth would be expected to be during a procedure or consultation.

The PAC (b) was placed where required for a particular setup. In the picture above, PAC P1 is placed in the ‘ideal’ position, elevated from the floor within a metre of the aerosol droplet generator and detector.

a – APS; b –PAC (P1); c – Hot wire anemometer; d – Flowmeter; e – Aerosol droplet outflow on Sonny; f – Nebuliser


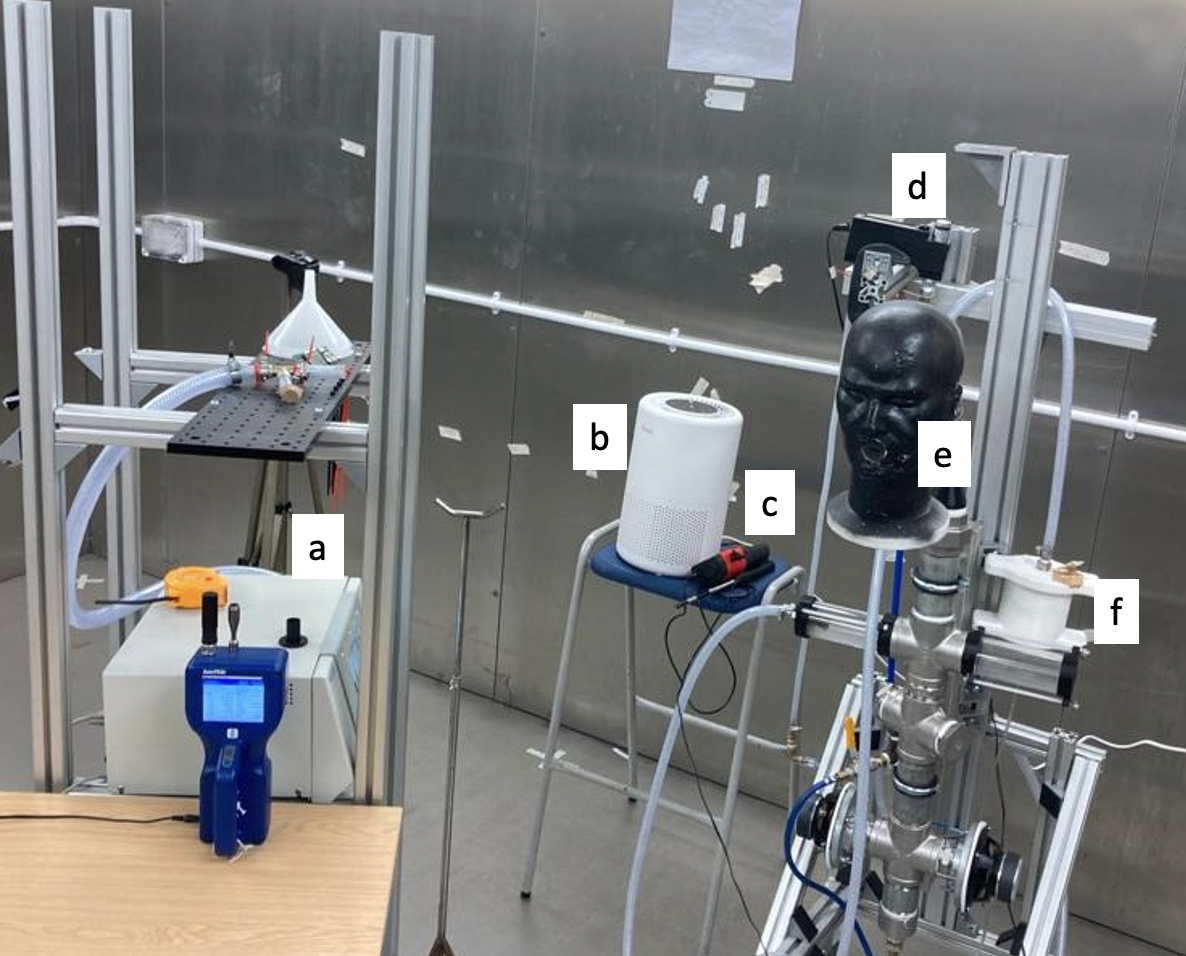


SI Figure S1: Aerosol droplet generator and detector setup. a – Aerodynamic Particle Sizer; b – Air PAC (P1); c – Hot wire anemometer; d – Flowmeter; e – Aerosol droplet outflow on Sonny; f – Nebuliser

### Portable Air Cleaners

P1 (Core 200S Smart True HEPA Air Purifier, Arovast Corporation, CA, USA), measuring 205 x 205 x 320 mm, running at 37 W rated power; and a larger unit, P2 (LV-H133 Tower True HEPA Air Purifier, Arovast Corporation, CA, USA), measuring 318 x 318 x 600 mm, running at rated power. When switched on, devices P1 and P2 were set to medium (setting 2 of 3), which resulted in an approximate air flow of 65 m^3^/hour and 130 m^3^/hour respectively. This flow rate was calculated for each device by multiplying the outlet cross sectional area by the outlet air velocity (measured from hot wire anemometer measurements taken at the outlet of each device). The medium flow setting was chosen after consultation with medical professionals, who indicated that while the high flow setting (60 dB and 56 dB at 0.5 m for P1 and P2 respectively) was too noisy to be used during all conversations with patients, the medium flow setting (47 dB and 42 dB at a distance of 0.5 m for P1 and P2 respectively) was acceptable.

### Laser Measurements

﻿Aerosol droplet dynamics were investigated via 2D PIV, a laser based, non-intrusive optical technique, enabling measurements of instantaneous velocity vector fields by correlating the displacement of aerosol droplets across a laser plane during a time interval Δt. The technique had been validated in a previous study for the capturing of aerosol droplets(1). The system set up is represented in SI Figure S2, where the positions of the camera and laser with respect to the aerosol droplet outflow are represented. Measurements were carried out on 2 outflow jet cross sections (coronal and sagittal plane with respect to Sonny), bisecting the centre of the outflow orifice (SI Figure S2iii).


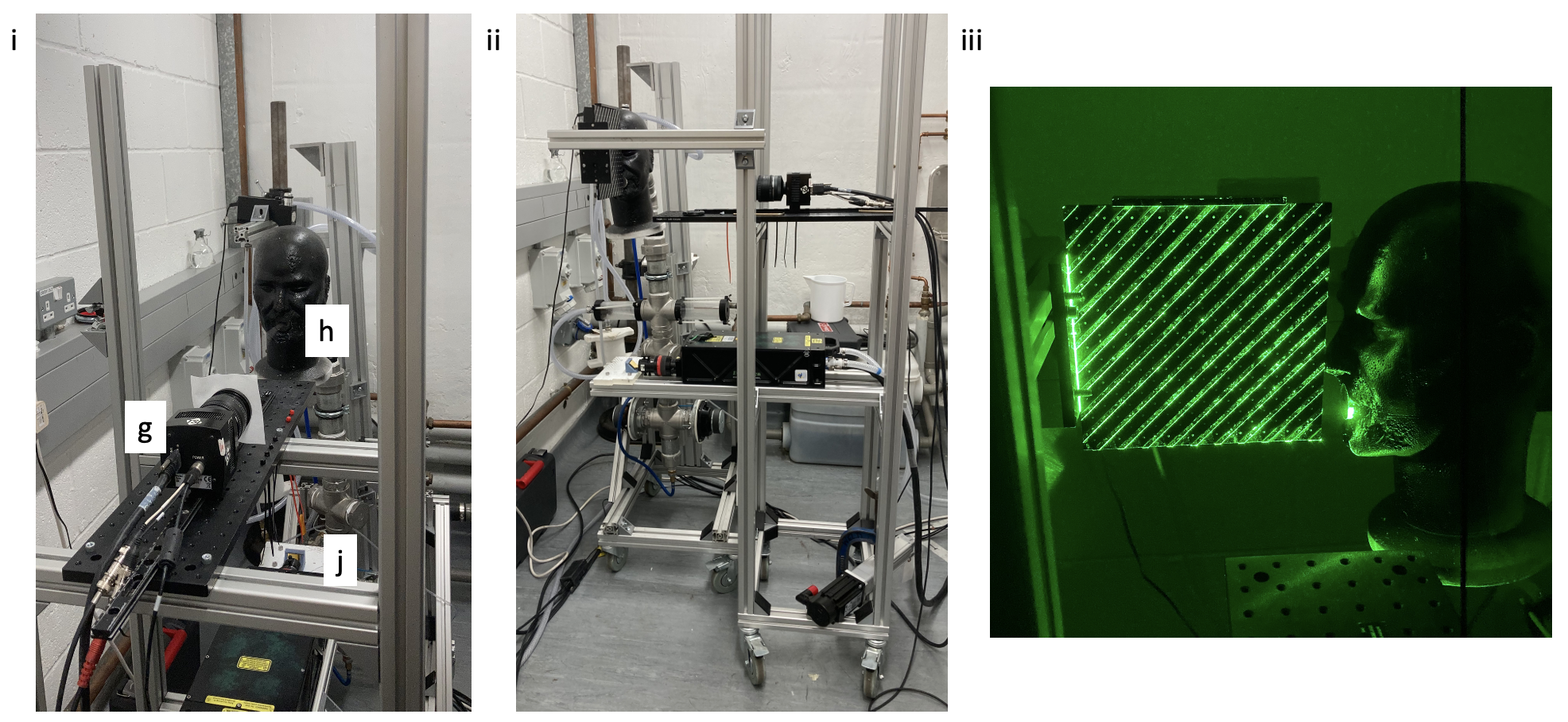


SI Figure S2: PIV setup. i – coronal plane setup. ii – sagittal plane setup. iii – laser sheet calibration for sagittal plane. g – high speed camera; h – aerosol droplet outflow on Sonny; j – dual cavities YAG laser

﻿A cylindrical lens laser (dual cavities YAG laser, 70 mJ -x2- at 15 Hz, 532 15 nm, 1.5 mm maximum laser thickness, (j)) illuminated the planar measurement region. A high-speed camera (TSI PowerView™ Plus 4MP, (g)) was used to capture the instantaneous positions of the aerosol droplets, with a LaserPulse™ Synchronizer (Model 610036, TSI Inc., US, with 1 ns of time resolution) ensuring correct timing between the laser pulses and the camera. A time interval (∆t) between consecutive frames of 100 µs was used; this allowed identification and quantification of the aerosol droplet movement. The velocity vectors from the PIV images were calculated through Insight 4G™ (TSI Inc., US), using an adaptive correlation algorithm with a final spatial resolution of 400 µm. The acquired images were processed with a recursive Nyquist grid (with an interrogation region varying from 64×64 to 32×32 pixels with an overlap of 50%), plus a fast Fourier transform correlation. Tecplot™ (Tecplot 26 Inc., US) was used to compute and visualise the streamlines.

﻿﻿The coronal plane PIV images (SI Figure S3) showed how the cross section of the jet changed as the distance from the orifice on Sonny increased, with the jet taking a rectangular shape to begin with, longer along the horizontal axis than the vertical), translating into a more square cross section, and then taking another rectangular shape, but now with a longer vertical axis than horizontal.


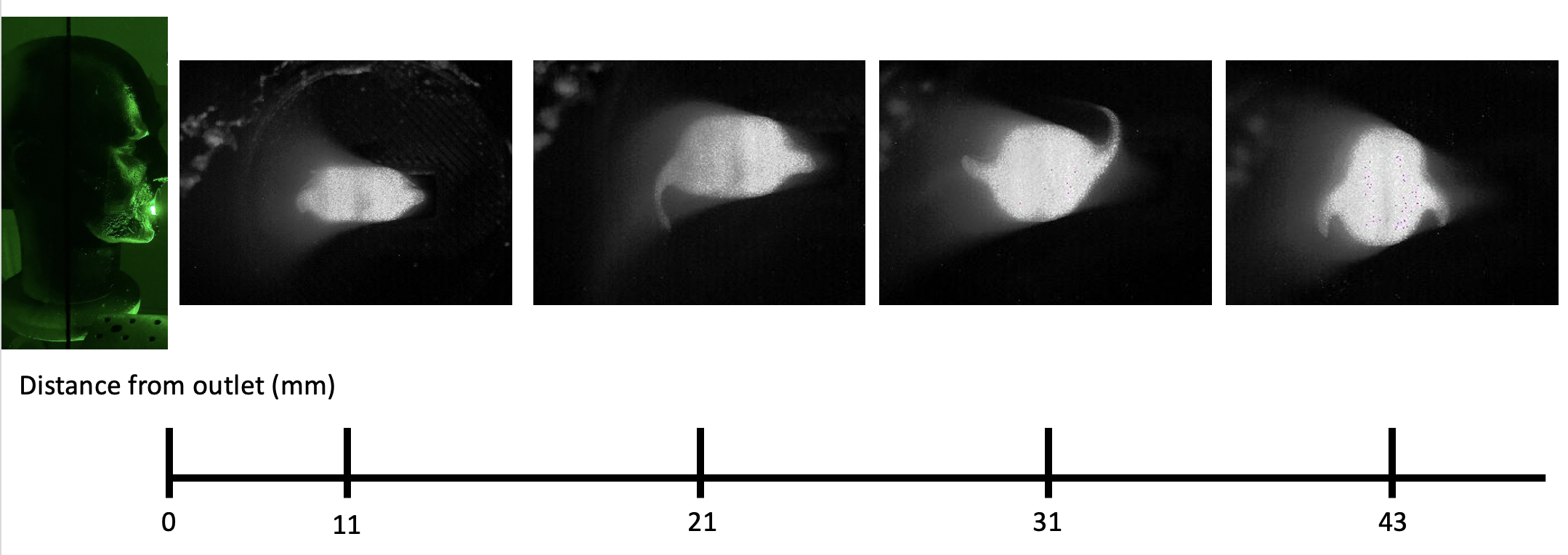


SI Figure S3: Coronal plane Particle Image Velocimetry images. These images show the how the coronal cross section of the outflow of aerosol droplets varies as the distance from the orifice increases.

The sagittal plane PIV images (SI Figure S4) enabled the measurement of the axial velocity of the aerosol droplets of the emerging jet. The mean velocity of the jet across the analysed area was 0.45 m/s, with a peak velocity of 1.19 m/s.


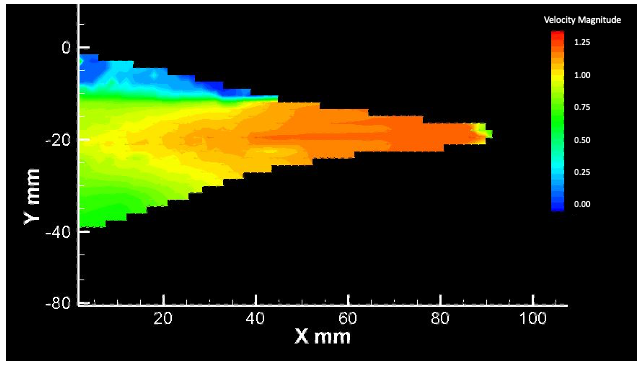


SI Figure S4: Sagittal plane Particle Image Velocimetry.

### Validation of experimental protocol

Air samples starting from different levels of FSSAC were taken for various mitigation setups, and the resultant decay constants and 𝜆 calculated. Three broad categories of FSSAC were investigated - full, medium and low initial steady state. These were obtained with different combination of pressure and PAC settings in droplet production, i.e., Sonny. Once FSSAC was achieved, the supply to the nebuliser was switched off, the mitigations settings were changed to those required for a particular decay analysis, and decay constants and 𝜆 were calculated according to Equations 1 and 2. Mitigations used for this validation analysis were: NP; P1_i_; P2_i_.

#### Full, medium, and low filling steady states

Full steady state was attained with a constant pressure supply of 1 bar throughout seeding, Medium steady state was achieved by only switching on the pressure supply to the nebuliser for 10 s every 120 s, with mitigation P1 running at a medium setting throughout, regardless of the decay analysis mitigation setup. Low steady state entailed the same 10 s of pressure supply to the nebuliser every 120s, but with both P1 and P2 mitigations running on the high setting.

Low, medium and full FSSACs were reached for each of 3 mitigations (NP, P1_i_, P2_i_). The FSSAC, 𝜆, and the decay rate for each setup and FSSAC type are shown in SI Table 1. The droplet source was then switched off and the aerosol concentration decay was measured. These results were then normalised with the initial aerosol concentration at the start of each aerosol droplet decay for each configuration for comparison (SI Figure S5i). The error bars in SI Figure S5i indicate the variability between experiments run with the same mitigation setup but for different FSSAC. Resultant 𝜆 across FSSACs for each mitigation ranged from 21.0 min (no mitigation, full FSSAC) to 3.3 min (P2_ideal_, low FSSAC) (SI Figure S5ii).

| Steady State Category | Mitigation | Filling Steady State Aerosol Concentration (#/cm^3^) | 𝜆 (min) | *b* (min^-1^) |
| --- | --- | --- | --- | --- |
| Low | NP | 51.8 ± 0.8 | 14.8 | -0.0468 |
|  | P1_i_ | 50.5 ± 0.6 | 4.7 | -0.1487 |
|  | P2_i_ | 51.2 + 1.4 | 3.3 | -0.2077 |
| Medium | NP | 245.9 ± 24.5 | 16.1 | -0.0428 |
|  | P1_i_ | 258.2 ± 28.5 | 6.4 | -0.1078 |
|  | P2_i_ | 226.2 ± 15.2 | 4.3 | -0.1621 |
| Full | NP | 1355.3 ± 20.0 | 21.0 | -0.0339 |
|  | P1_i_ | 666.2 ± 7.9 | 8.0 | -0.0879 |
|  | P2_i_ | 370.1 ± 4.6 | 5.6 | -0.1236 |

SI Table S1: Steady state variation. Aerosol concentrations are given as mean of 1 min sample taken every 2 min for 10 min, ± standard deviation. 𝜆 is the half-life for the decay of aerosol droplets, b is the decay constant for this decay. The ± variation for filling steady state aerosol concentration reflects the standard deviation of measurements taken that satisfied the requirements for steady state.


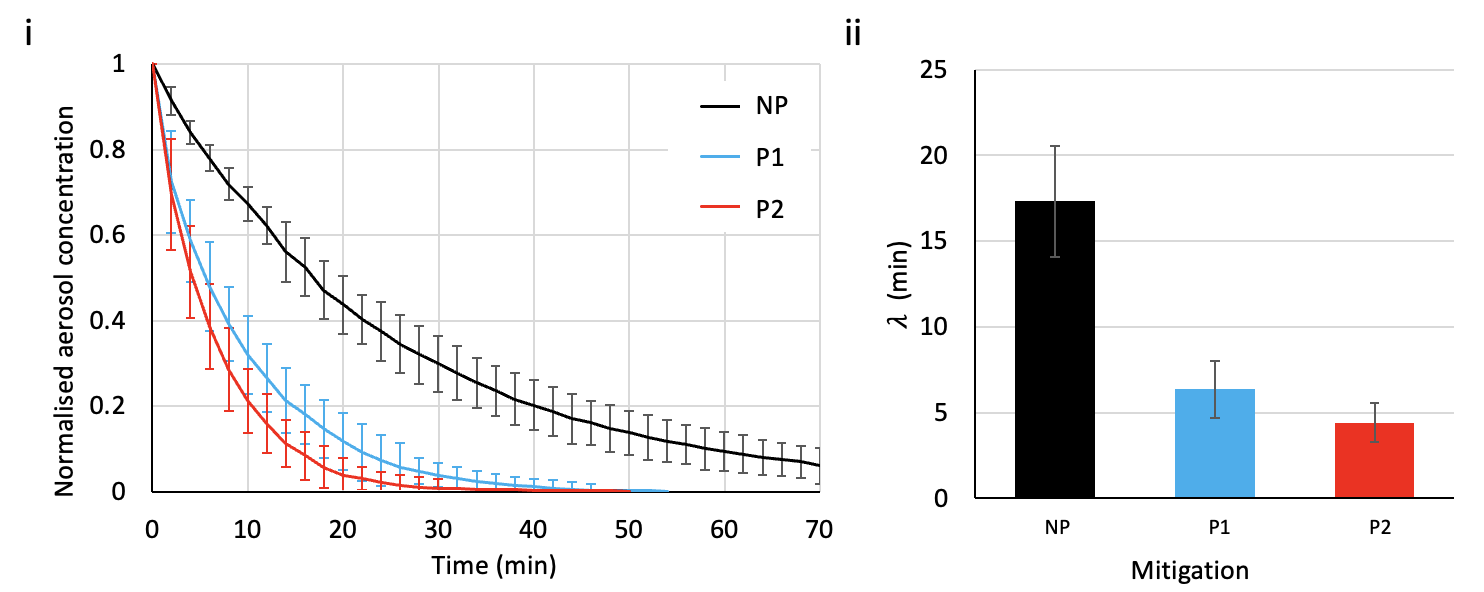


SI Figure S5: Variation of decay with filling steady state aerosol concentration (FSSAC). (i) Mean reduction of normalised aerosol concentration with time for various mitigation strategies starting from full, medium, and low FSSACs. (ii) Mean half-lives (𝜆) for each mitigation type across various FSSACs. Error bars in both graphs are standard deviations across normalised means for the three FSSACs for each mitigation type. NP – PAC; P1 – small PAC on medium setting; P2 – large PAC on medium setting. These data were captured from experiments in a UCL laboratory (R1).

SI Figure S5i clearly shows that the protocol implemented in the experiments is only marginally affected by the FSSAC. In fact, the variation between curves of normalised aerosol concentrations for different mitigations (NP, P1, P2) is less than the variability due to the initial aerosol concentration for a given mitigation (indicated by the error bars in SI Figure S5i), i.e., even with the variability of different FSSAC taken into account, the distinction between mitigation strategies is clear. Additionally, SI Table S1 shows that a higher initial FSSAC consistently results in a longer 𝜆. Therefore, the proposed experimental protocol increases the signal:noise error and provides a conservative estimate of air cleaning time.

#### Repeatability Experiments

As time in the hospital was limited, only 1 run of each setup would be undertaken, in order to test for a wide variety of mitigation types and configurations. To increase confidence that a single run would be representative of a specific mitigation type/configuration and would contribute towards valid comparisons between the various setups, multiple runs of the same mitigation type/configuration were run in the UCL lab, to see the variability of the decay constant and half-life for each setup.

Each of the chosen two setups – no PAC with closed mechanical ventilation and PAC P1 with closed mechanical ventilation - was run 3 times, with the results shown in SI Table S2. For the No PAC-closed vents setup, this gave a decay constant of 0.0316 - 0.0330 and a half-life of 21.2 – 22.1 min, i.e., a range of 4.4% and 4.2% respectively. For the P1 PAC-closed vents setup, this resulted in a decay constant of 0.0865 - 0.0911 and a half-life of 7.8 – 8.1 min, i.e., a range of 5.3% and 3.8% respectively.

| Mitigation | Run | Baseline aerosol concentration (#/cm^3^) | Filling steady state aerosol concentration (#/cm^3^) | *a* (#/cm^3^) | *b (min^-1^)* | *c_0_* (#/cm^3^) | 𝜆 (min) |
| --- | --- | --- | --- | --- | --- | --- | --- |
| No PAC | 1 | 6.62 | 1255.98 | 1205 | 0.0330 | 9.61 | 21.2 |
| No PAC | 2 | 7.39 | 1289.48 | 1299 | 0.0316 | 5.75 | 22.1 |
| No PAC | 3 | 5.35 | 1383.04 | 1341 | 0.0329 | 9.80 | 21.3 |
| P1 | 1 | 6.02 | 620.50 | 611 | 0.0903 | 8.40 | 7.8 |
| P1 | 2 | 6.72 | 755.11 | 724 | 0.0911 | 11.72 | 7.8 |
| P1 | 3 | 5.96 | 595.42 | 579 | 0.0865 | 6.61 | 8.1 |

SI Table S2: Experimental Constants and Baseline and Filling Steady State aerosol concentrations for Experimental Repeatability study. The ‘Baseline aerosol concentration’ is defined as the mean of 5 consecutive pre-seeding measurements of aerosol concentration. The ‘Filling steady state aerosol concentration’ was defined as when the aerosol concentration readings remained constant ±10% for 5 consecutive readings.

### Experimental Baseline and Filling Steady State Aerosol Concentrations

#### UCL Laboratory Baseline and Filling Steady State Aerosol Concentrations

| Mitigation | Mitigation Location | Mechanical Ventilation | Baseline aerosol concentration (#/cm^3^) | Filling steady state aerosol concentration (#/cm^3^) |
| --- | --- | --- | --- | --- |
| No PAC | n/a | Closed | 7.36 | 1355.92 |
| No PAC | n/a | Open | 8.14 | 530.48 |
| P1 | ‘Ideal’ | Closed | 6.47 | 660.85 |
| P1 | ‘Ideal’ | Open | 5.92 | 454.76 |
| P1 | In corner | Closed | 4.69 | 643.96 |
| P1 | Under table | Closed | 6.19 | 730.11 |
| P1 | By door | Closed | 6.35 | 1062.9 |
| P1+P2 | ‘Ideal’ | Closed | 7.12 | 313.42 |
| P2 | ‘Ideal’ | Closed | 4.12 | 341.97 |
| P2 | In corner | Closed | 5.03 | 349.05 |

SI Table S3: Baseline and Filling Steady State aerosol concentrations for UCL Laboratory (R1). The ‘Baseline aerosol concentration’ is defined as the mean of 5 consecutive pre-seeding measurements of aerosol concentration. The ‘Filling steady state aerosol concentration’ was defined as when the aerosol concentration readings remained constant ±10% for 5 consecutive readings.

#### Hospital Experiment Baseline and Filling Steady State Aerosol Concentrations

| Mitigation | Mitigation Location | Mechanical Ventilation | Baseline aerosol concentration (#/cm^3^) | Filling steady state aerosol concentration (#/cm^3^) |
| --- | --- | --- | --- | --- |
| No PAC | n/a | No PAC | 8.75 | 1674.75 |
| P1 | ‘Ideal’ | P1 | 8.34 | 445.11 |
| P1 | Under table | P1 | 6.38 | 786.00 |
| P1+P2 | ‘Ideal’ | P1+P2 | 6.92 | 238.10 |
| P2 | ‘Ideal’ | P2 | 4.52 | 380.33 |

SI Table S4: Baseline and Filling Steady State aerosol concentrations for UCLH Consultation Room (R2). The ‘Baseline aerosol concentration’ is defined as the mean of 5 consecutive pre-seeding measurements of aerosol concentration. The ‘Filling steady state aerosol concentration’ was defined as when the aerosol concentration readings remained constant ±10% for 5 consecutive readings.

| Mitigation | Mitigation Location | Mechanical Ventilation | Baseline aerosol concentration (#/cm^3^) | Filling steady state aerosol concentration (#/cm^3^) |
| --- | --- | --- | --- | --- |
| No PAC | n/a | No PAC | 8.29 | 2340.66 |
| P1 | ‘Ideal’ | P1 | 7.83 | 485.72 |
| P1 | Under table | P1 | 5.46 | 836.26 |
| P1+P2 | ‘Ideal’ | P1+P2 | 6.31 | 271.84 |
| P2 | ‘Ideal’ | P2 | 4.71 | 412.38 |

SI Table S5: Baseline and Filling Steady State aerosol concentrations for UCLH Procedure Room (R3). The ‘Baseline aerosol concentration’ is defined as the mean of 5 consecutive pre-seeding measurements of aerosol concentration. The ‘Filling steady state aerosol concentration’ was defined as when the aerosol concentration readings remained constant ±10% for 5 consecutive readings.

### Experimental Constants

Each experiment resulted in setup and environment specific constants to fit the equation:

$$c=ae^{-bt}+c_{0}$$

where *c* = aerosol concentration (#/cm^3^), *t* = time (minutes), and *a*, *b* and *c_0_* are constants specific to each decay curve (in the units #/cm^3^, min^-1^, and #/cm^3^ respectively). These constants were then used to calculate a half-life (𝜆, min) of aerosol concentration during cleaning as follows:

$$\lambda=\frac{ln\left( \frac{a-c_{0}}{2a} \right)}{b}$$

The resultant constants and 𝜆 are listed below.

#### Experimental Validation Constants

| Mitigation | Steady State | *a* (#/cm^3^) | *b (min^-1^)* | *c_0_* (#/cm^3^) | 𝜆 (min) |
| --- | --- | --- | --- | --- | --- |
| No PAC | Low | 29.1 | 0.0467 | 8.69 | 22.4 |
| P1 | Low | 41.9 | 0.1487 | 5.99 | 5.7 |
| P2 | Low | 46.2 | 0.2078 | 5.05 | 3.9 |
| No PAC | Medium | 174.2 | 0.0431 | 10.79 | 17.6 |
| P1 | Medium | 223.0 | 0.1078 | 8.48 | 6.8 |
| P2 | Medium | 226.5 | 0.1622 | 7.53 | 4.5 |
| No PAC | Full | 1324.0 | 0.0325 | 8.44 | 21.6 |
| P1 | Full | 652.9 | 0.0877 | 7.79 | 8.0 |
| P2 | Full | 350.2 | 0.1236 | 1.39 | 5.6 |

SI Table S6: Experimental Constants for Experimental Validation Study.

#### UCL Laboratory Experiment Constants

| Mitigation | Mitigation Location | Mechanical Ventilation | *a* (#/cm^3^) | *b (min^-1^)* | *c_0_* (#/cm^3^) | 𝜆 (min) |
| --- | --- | --- | --- | --- | --- | --- |
| No PAC | n/a | Closed | 1323.8 | 0.0325 | 8.44 | 21.6 |
| No PAC | n/a | Open | 546.5 | 0.1296 | 0.02 | 5.4 |
| P1 | ‘Ideal’ | Closed | 652.9 | 0.0877 | 7.79 | 8.0 |
| P1 | ‘Ideal’ | Open | 457.2 | 0.1821 | 2.39 | 3.8 |
| P1 | In corner | Closed | 631.2 | 0.0778 | 4.98 | 9.0 |
| P1 | Under table | Closed | 722.2 | 0.0609 | 5.91 | 11.5 |
| P1 | By door | Closed | 1084.0 | 0.0681 | 5.44 | 10.3 |
| P1+P2 | ‘Ideal’ | Closed | 312.3 | 0.2064 | 1.77 | 3.4 |
| P2 | ‘Ideal’ | Closed | 350.2 | 0.1236 | 1.39 | 5.6 |
| P2 | In corner | Closed | 351.1 | 0.1391 | 2.59 | 5.0 |

SI Table S7: Experimental Constants for Aerosol Clearing in UCL Laboratory (R1)

#### Hospital Experiment Constants

| Mitigation | Mitigation Location | *a* (#/cm^3^) | *b (min^-1^)* | *c_0_* (#/cm^3^) | 𝜆 (min) |
| --- | --- | --- | --- | --- | --- |
| No PAC | n/a | 1456.2 | 0.0174 | 248.41 | 50.5 |
| P1 | ‘Ideal’ | 443.7 | 0.0720 | 1.52 | 9.7 |
| P1 | Under table | 766.1 | 0.0536 | 1.29 | 13.0 |
| P1+P2 | ‘Ideal’ | 238.5 | 0.1641 | 0.68 | 4.2 |
| P2 | ‘Ideal’ | 383.4 | 0.1024 | 0.20 | 6.8 |

SI Table S8: Experimental Constants for Aerosol Clearing in UCLH Consultation Room (R2)

| Mitigation | Mitigation Location | *a* (#/cm^3^) | *b (min^-1^)* | *c_0_* (#/cm^3^) | 𝜆 (min) |
| --- | --- | --- | --- | --- | --- |
| No PAC | n/a | 2156.1 | 0.0085 | 85.35 | 85.4 |
| P1 | ‘Ideal’ | 492.5 | 0.0527 | 13.16 | 13.2 |
| P1 | Under table | 773.8 | 0.0557 | 12.75 | 12.8 |
| P1+P2 | ‘Ideal’ | 276.2 | 0.1344 | 5.19 | 5.2 |
| P2 | ‘Ideal’ | 413.2 | 0.0922 | 7.62 | 7.6 |

SI Table S9: Experimental Constants for Aerosol Clearing in UCLH Procedure Room (R3)

### Aerosol Size Distributions

Aerosol size distributions were captured during all experiments, with representative measurements presented in SI Figure S6 and SI Figure S7.


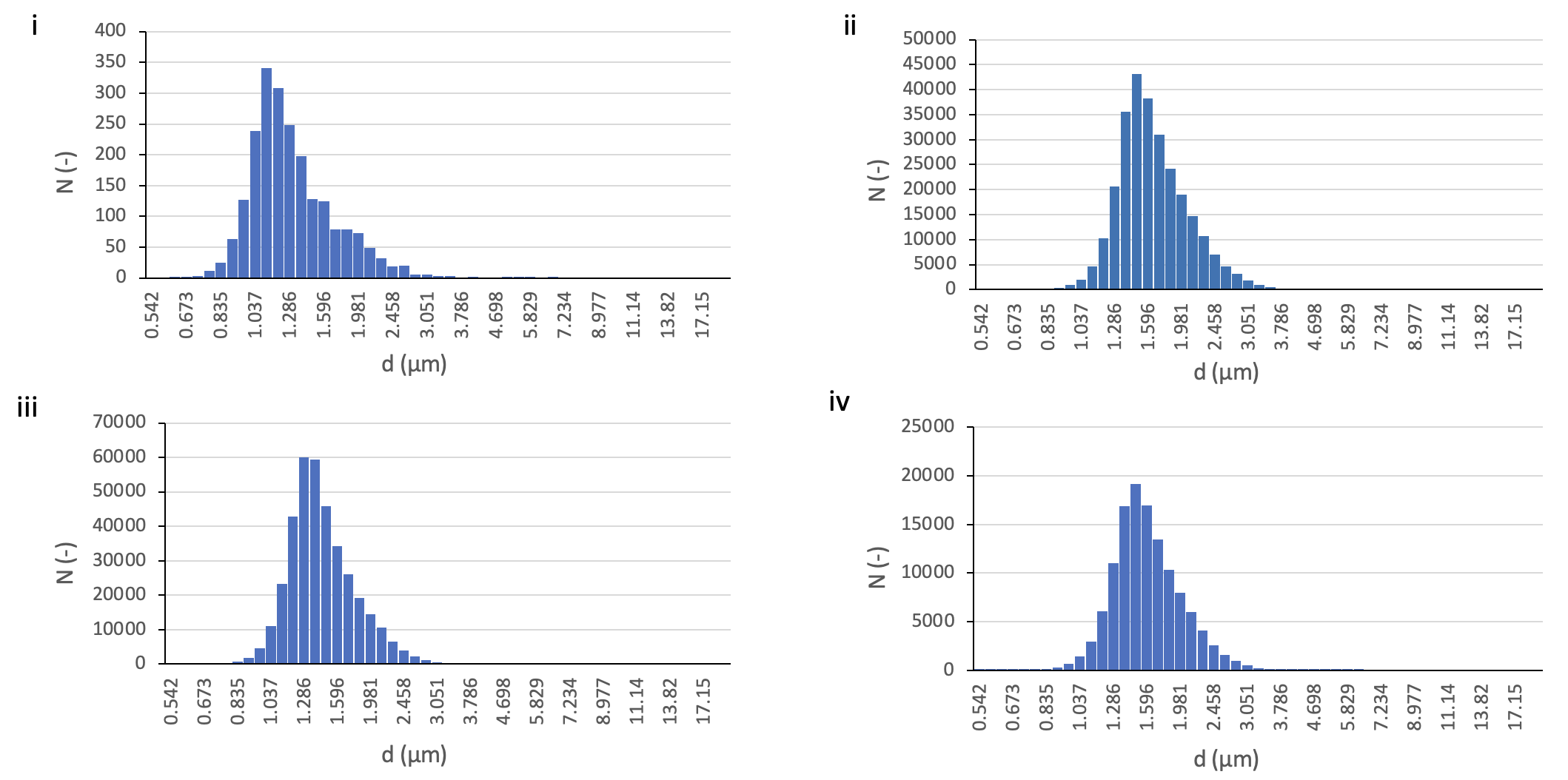


SI Figure S6: Aerosol Size Distributions A. i – smallest mean aerosol diameter distribution measurements taken across all experiments, taken at emptying steady state aerosol concentration for P1_i_ in R1 with open vents; ii - largest mean aerosol diameter distribution measurements taken across all experiments, taken at filling steady state aerosol concentration (FSSAC) for P2_c_ in R1 with closed vents. iii – typical aerosol size distribution for open vents, taken at FSSAC in R1 with no mitigation (NP). iv - typical aerosol size distribution for closed vents, taken at FSSAC in R1 with no mitigation (NP).


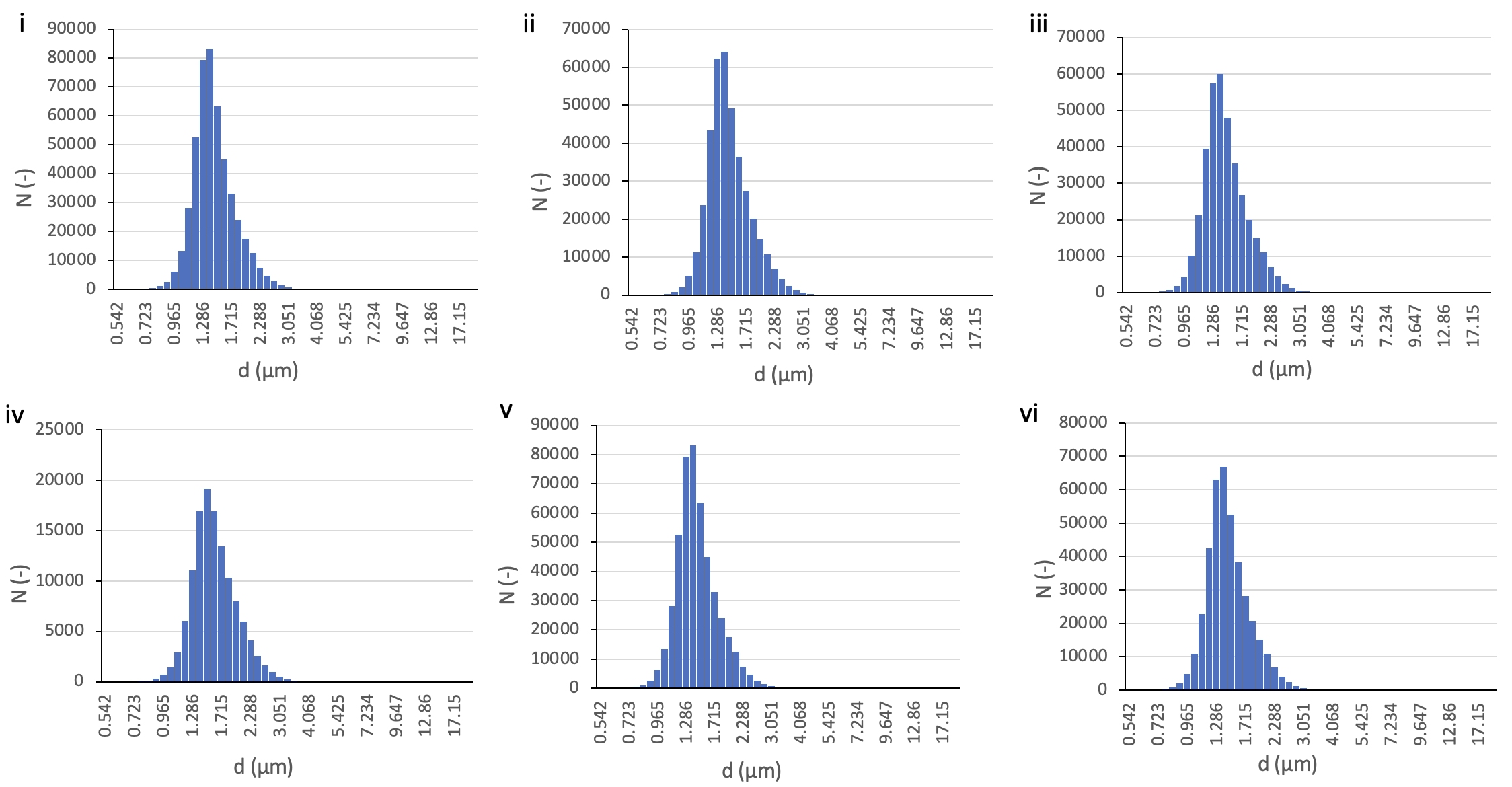


SI Figure S7: Aerosol Size Distributions B. All distributions in this figure were taken at filling steady state aerosol concentration, with closed vents. i – typical aerosol size distribution for no mitigation (NP), taken in R2. ii - typical aerosol size distribution for P1 mitigation, taken in R2. iii - typical aerosol size distribution for P2, taken in R2. iv - typical aerosol size distribution for R1 (no mitigation). v – typical aerosol size distribution for R2 (no mitigation). vi – typical aerosol size distribution for R3 (no mitigation).

### Filling rate

Filling rates for the plots in Figure 5 in the manuscript were estimated by applying a line of best fit to the aerosol concentration filling curves from the experiments in R3 for the NP and P1i setups. The equation for the line of best fit was:

$$c=a\left( 1-e^{-bt} \right)$$

(SI-1)

where c = aerosol concentration (cm^-3^), t = time (min), a = constant (cm^-3^) and b is the filling rate (min^-1^)). For the NP configuration, a = 2788 cm^-3^ and b = 0.0275 min^-1^. For the P1i configuration, a = 668 cm^-3^ and b = 0.043 min^-1^.

## References

1. Ho KMA, Davies H, Epstein R, Bassett P, Hogan Á, Kabir Y, et al. Spatiotemporal droplet dispersion measurements demonstrate face masks reduce risks from singing. Sci Rep. 2021;11(1):1–11.
